# Improved PCR diagnostics using up-to-date *in silico* validation: An F-gene RT-qPCR assay for the detection of all four lineages of peste des petits ruminants virus

**DOI:** 10.1016/j.jviromet.2019.113735

**Published:** 2019-12

**Authors:** John Flannery, Paulina Rajko-Nenow, Hannah Arnold, Erik van Weezep, Piet A. van Rijn, Chanasa Ngeleja, Carrie Batten

**Affiliations:** a The Pirbright Institute, Ash Road, Pirbright, Woking, Surrey, GU24 0NF, United Kingdom; b Department of Virology, Wageningen Bioveterinary Research (WBVR), Lelystad, the Netherlands; c Department of Biochemistry, North West University, Potchefstroom, South Africa; d Centre for Infectious Diseases and Biotechnology, Tanzania Veterinary Laboratory Agency, Dar es Salaam, Tanzania

**Keywords:** RT-qPCR, PPRV, *In silico*, Rapid detection

## Abstract

Peste des petits ruminants (PPR) is a globally significant disease of small ruminants caused by the peste des petits ruminants virus (PPRV) that is considered for eradication by 2030 by the United Nations Food and Agriculture Organisation (FAO). Critical to the eradication of PPR are accurate diagnostic assays. RT-qPCR assays targeting the nucleocapsid gene of PPRV have been successfully used for the diagnosis of PPR. We describe the development of an RT-qPCR assay targeting an alternative region (the fusion (F) gene) based on the most up-to-date PPRV sequence data. *In silico* analysis of the F-gene RT-qPCR assay performed using PCRv software indicated 98% sensitivity and 100% specificity against all PPRV sequences published in Genbank. The assay indicated the greatest *in silico* sensitivity in comparison to other previously published and recommended PPRV RT-qPCR assays. We evaluated the assay using strains representative of all 4 lineages in addition to samples obtained from naturally and experimentally-infected animals. The F-gene RT-qPCR assay showed 100% diagnostic specificity and demonstrated a limit of detection of 10 PPRV genome copies per μl. This RT-qPCR assay can be used in isolation or in conjunction with other assays for confirmation of PPR and should support the global efforts for eradication.

## Highlights

• Designed F-gene RT-qPCR using all full-genomes available on genbank.
• Performed *in silico* evaluation of existing and new PPRV RT-qPCR assays.
• F-gene RT-qPCR assay shows the greatest *in silico* performance.
• The assay demonstrates excellent diagnostic and analytical sensitivity.
• The assay may be useful during the global PPR eradication campaign.

## 1 Introduction

Peste des Petits Ruminants (PPR) is a viral disease of small ruminants which is a serious threat to food security in countries reliant on small-scale agriculture such as those across the developing world (Banyard et al., 2010). PPR has spread globally since it was described in the early 20^th^ century in Côte d’Ivoire (Gargadennec and Lalanne, 1942) and the disease is present in Africa, Europe and Asia. The global burden of this disease (estimated up to $2.1 USD annually) is well recognised (Baron et al., 2011), hence PPR is considered for eradication by 2030 by the United Nations Food and Agriculture Organisation (FAO) and the World Organisation for Animal Health (OIE) (FAO, 2015). The PPR eradication campaign will benefit food sustainability across the developing world as did the eradication of rinderpest (a disease of large ruminants caused by a closely-related morbillivirus, rinderpest virus) in 2011. It is anticipated that the successful eradication of PPR will come about through global collaboration, accurate diagnostic assays and effective vaccination campaigns (Baron et al., 2017).

PPR has high mortality in goats (between 50–90%) (Parida et al., 2015) and clinical signs can include pyrexia up to 41 °C, erosive lesions in the oral cavity which leads to excessive salivation, mucopurulent ocular and nasal discharge, coughing, depression and diarrhoea. Milder strains have low morbidity which can lead to the disease being overlooked or misdiagnosed in the field. It is critical that clinical diagnosis is supported by laboratory diagnosis, of which molecular assays present the most sensitive technique. These assays are used in most diagnostic laboratories and a number of conventional and real-time RT-PCR assays are cited in the OIE (World Organisation for Animal Health) terrestrial manual (Libeau and Baron, 2013).

PPR is caused by Small Ruminants Morbillivirus which is more-often referred to as Peste des Petits Ruminants virus (PPRV). PPRV is a Morbillivirus within the family *Paramyxoviridae* and has a linear negative-stranded RNA that encodes for 6 structural proteins and two non-structural proteins (Parida et al., 2015). The fusion, nucleocapsid and hemagglutinin proteins encoded by the F-, N- and H-genes, respectively, have been used to classify PPRV into 4 distinct lineages which correspond well with their geographical distribution (Parida et al., 2015). As the PPRV N-gene lies close to the viral promoter, it is the most abundant protein produced during replication (Kumar et al., 2014), and as such has been used as a target for some of the most widely-used RT-qPCR assays to date (Batten et al., 2011; Kwiatek et al., 2010; Polci et al., 2015).

In spite of various control programmes, PPR (especially that caused by lineage IV viruses) continues to spread into new regions, with the 2018 Bulgarian outbreak representing the first European incursion of the disease (World Organisation for Animal Health, 2018). As with other viral transboundary diseases such as Bluetongue, Schmallenberg and Avian Influenza, the continued spread of the causative virus has the potential to generate mutations which may undermine the sensitivity of the in-use molecular assays (Hoffmann et al., 2012; Hofmann et al., 2008; Maan et al., 2011; Tong et al., 2018). A number of mismatches in the primer/probe binding site can yield false-negative results while false positive results can arise from cross-reactivity with nucleic acid of other organisms present in the sample (van Weezep et al., 2019). It has been reported that a number of recent PPRV sequences contain mismatches which, through *in silico* analysis, suggests that these strains may not be detected using existing PPRV RT-qPCR assays (van Rijn et al., 2018). It is therefore important that the recommended assays are appropriate for the current situation given the potential for new PPRV strains to arise.

Recently, a software tool named PCRv was developed to facilitate the *in silico* validation of molecular-based detection methods (van Weezep et al., 2019). This program combines a number of freely-available software packages to generate a report which indicates the sensitivity and specificity of the designed assay against all publically available sequences in Genbank. As PPR has continued to spread since the assays specified in the OIE terrestrial manual were published, we aimed to assess the suitability of these assays in comparison with newly-designed PPRV RT-qPCR assays. Based on the results generated using PCRv, we selected the primers/probe targeting the F-gene for further laboratory evaluation. The F-gene RT-qPCR assay has undergone extensive *in silico* and laboratory evaluation alongside an OIE recommended PPRV RT-qPCR assay and should be considered as an appropriate confirmatory molecular assay.

## 2 Material and methods

### 2.1 Design of the RT-qPCR assay

Sixty seven full-genome PPRV sequences were obtained from Genbank and were aligned using MEGA6: Molecular Evolutionary Genetics Analysis Version 6.0. The sequences represented all lineages of PPRV: lineage I (3 sequences), lineage II (8 sequences), lineage III (6 sequences) and lineage IV (50 sequences). A multiple alignment was then performed for each coding region: the nucleoprotein (N-gene), phosphoprotein (P-gene), matrix protein (M-gene), fusion protein (F-gene), haemagglutinin (H-gene) and large polymerase protein (L-gene). Primers and probes were designed for each region using Primer express Software (ThermoFisher Scientific, Paisley, UK). Each assay was designed so to have a maximum of two degenerate bases for each single primer but without any degenerate bases for each probe. Primers and probes were evaluated for their Tm, hairpins, and primer–primer/primer–probe interactions using the Primer express software and OligoAnalyzer 3.0 (Integrated DNA Technologies, http://www.idtdna.com/analyzer/applications/oligoanalyzer/). All probes designed were labelled as 5’-FAM and 3’-MGB (minor groove binder) and are listed in Table 1.

**Table 1:** *In silico* evaluation of PPRV RT-qPCR assays using PCRv software.

PCR	PPRV region	Forward primer sequence (5’-3’)	Reverse primer sequence (5’-3’)	Probe Sequence (5’-3’)	Author	FICS performance	Sensitivity	Specificity
1	F-gene	CATAGSACTGGCAGCTTGCA	GAGCCCTGGGTTGATTTTRG	CTTGTCACATTAATATGCTG	This study	4.6	98.0	100
2	H-gene	CGAKCATGCNATCGTGTA	CTCTATCCTCADAGAGAGAGGRTTG	TCGCTCATCATCTTAC	This study	3.1	72.2	100
3	L-gene	ATCGGGATGTCYCCTATAGAAA	AAGRGGATCAGTGTTCCATGA	TCACACATTACATCAAGGG	This study	4.5	96.3	100
4	M-gene	CACMGGGAAAATGAGCAARA	CCCGCCARAGATATCGRTT	CCTCCATGCACAGCT	This study	2.5	99.1	100
5	N-gene	AGTCCGGRTTGACCTTTGCA	GTTCTCAAACCAGTTGATCCTTTTC	CACGTGGTGCTGATT	This study	2.6	94.8	100
6	P-gene	GAAGAGATTGAAGGACTCGAGGAT	CCACATCGCTGTYGTCAGATC	CTGACTCTCTCGTGGTTC	This study	3.9	98.8	100
7	N-gene	CACAGCAGAGGAAGCCAAACT	TGTTTTGTGCTGGAGGAAGGA	CTCGGAAATCGCCTCGCAGGCT	Bao et al., 2008	5.0	46.9	100
8	N-gene	GAGTCTAGTCAAAACCCTCGTGAG	TCTCCCTCCTCCTGGTCCTC	CGGCTGAGGCACTCTTCAGGCTGC	Kwiatek et al., 2010	4.8	58.6	100
9	N-gene	AGAGTTCAATATGTTRTTAGCCTCCAT	TTCCCCARTCACTCTYCTTTGT	CACCGGAYACKGCAGCTGACTCAGAA	Batten et al., 2011	5.7	90.5	100
10	N-gene	CGCCTTGTTGAGGTAGTTCAAAGT	ATCAGCACCACGTGATGCA	CAGTCCGGGTTGACCT	Polci et al., 2015	3.0	90.6	100
11	N-gene	CACAGCAGAGGAAGCCAAACT	TGTTTTGTGCTGGAGGAAGGA	CTCGGAAATCGCCTCGCAGGCT	Ashraf et al., 2017	3.3	13.1	100
12	N-gene	AGTATCCGCCTTGTTGAGGT	TCTATTATTTCTCTGTTCTCAAACC	AGTCCGGGTTGACCTTTGCA	van Rijn et al., 2018	4.6	89.7	100

FICS Flagged internal control sequences.

### 2.2 *In silico* assessment of PPRV RT-qPCR assay

*In silico* assay assessment was performed as described (van Weezep et al., 2019). Briefly, 1445 PPRV full and partial sequences (Taxonomy ID: 31604) were downloaded from Genbank. Using the PCRv software, an alignment was made using ClustalW, Muscle, and Validatie software. By including the assay primers and probes outlined in Table 1, a conservation plot was prepared and from this, the *in silico* assay sensitivity was determined. To determine the *in silico* assay specificity, an alignment search was performed on the entire NCBI nucleotide database using the primers and probe, based on the Smith-Waterman algorithm. The PCRv program was used to determine whether certain primer/probe combinations could cross react with non-PPRV templates. To monitor the performance of PCRv, a set of flagged internal control sequences (FICS) was randomly added. A set of FICS consists of randomly-generated 3 kb sequences containing the assay primer and probe sequences in all possible combinations and orientations with an increasing number of mismatches to a maximum of 10 that could initiate amplification (van Weezep et al., 2019). The number of returned hits of control sequences (the FICS score) with an increasing number of mismatches was indicative for the sensitivity and accuracy of the alignment search. All assays designed in this paper targeting 6 PPRV structural proteins in addition to five N-gene assays (two specified in the OIE manual) were included in the *in silico* assessment (Table 1). To decide which assay would go forward for further evaluation, we applied selection criteria as follows: the assay must show an *in silico* specificity of 100%, an *in silico* sensitivity of >98% and yield the highest FICS score.

### 2.3 Automated RNA extraction

RNA was extracted using the KingFisher Flex (ThermoFisher) and the LSI™ Magvet Universal isolation kit (ThermoFisher) from 100 μl of sample material. Viral RNA was eluted into 80 μl of MagVet elution buffer which was stored at −20 °C until analysis using RT-qPCR.

### 2.4 Virus isolates and clinical specimens

Diagnostic sensitivity of the RT-qPCR assay was evaluated using samples which had been submitted to The Pirbright Institute, UK in its role as OIE reference laboratory for PPR. A total of 58 samples from numerous disease outbreaks in which PPRV was suspected were tested using the F-gene RT-qPCR assay alongside the in-house, ISO/IEC17025-accredited N-gene RT-qPCR assay described by Batten et al., 2011. The submitted samples consisted of ovine (n = 20) or caprine (n = 33): EDTA blood (n = 25), oral and faecal swabs (n = 12) and various tissues (n = 21) as shown in Table 3. EDTA blood, ocular and nasal swabs obtained from 4 goats that were experimentally-infected with PPRV (under project licence PL70/8833) over a period from 0 to 8 days post infection (dpi) were tested using the N-gene and F-gene RT-qPCR assays. The inocula for goats 1–4 were Ivory Coast/1989 (lineage I), Ghana/1978/1 (lineage II), Iran/2011 (lineage IV) and Tbilisi/Georgia/2016 (lineage IV), respectively. In addition, RNA extracted from PPRV isolates representing the 4 lineages (lineage I- IV) were tested by the F-gene RT-qPCR assay. Ten-fold dilutions (neat to 10^−6^ copies μl^-1^) were prepared in MagVet elution buffer and were analysed in triplicate using the F-gene RT-qPCR assay. Diagnostic specificity was evaluated using RNA extracted from a number of viruses that either show close genetic relationship with PPRV or cause a similar clinical diagnosis to PPR (Supplementary data). The most commonly-used PPRV vaccine strain (Nig75/1) was used as a positive control throughout.

### 2.5 RT-qPCR

Both the F-gene RT-qPCR assay and the N-gene RT-qPCR assay described by Batten et al., 2011 were performed using the Express One-Step Superscript qRT-PCR kit (LifeTechnologies, Paisley, UK). For each assay, 17 μl of one-step reaction mix was prepared using 1 × reaction mix, 400 nM forward and reverse primers, 200 nM probe, 0.4 μl Rox, and 2 μl of enzyme. Three microlitres RNA was used per well in a final volume of 20 μl. Cycling conditions were as follows: reverse transcription at 50 °C for 15 min and 95 °C for 20 s, and then 40 cycles of PCR, with each cycle consisting of 95 °C for 3 s and 60 °C for 30 s. RT-qPCR was performed on an Applied Biosystems 7500 Fast real-time PCR instrument (LifeTechnologies).

### 2.6 Assessment of efficiency, analytical sensitivity/ limit of detection

A PPRV ultramer was synthesized (148 bp dsDNA) by Integrated DNA Technologies with its sequence corresponding to 6993–7140 bp of Georgia/Tbilisi/2016 isolate (MF737202.1). Tenfold dilutions of the PPRV ultramer ranging from 10^7^ to 10^0^ copies μl^−1^ were prepared in MagVet elution buffer. The assay efficiency was determined as previously described (Ramakers et al., 2003). The limit of detection (LOD) was considered as the greatest dilution for which all 10 replicates tested positive as described by Forootan et al., 2017.

## 3 Results

### 3.1 *In silico* selection of RT-qPCR assay and comparison with published RT-qPCR assays

GenBank data files for all PPRV virus isolates (n = 1445) were downloaded from the NCBI taxonomy database and from this, a conservation plot was generated for 99 sequences that were in the area of the genome used for each RT-qPCR assay. The integrity of the downloaded NCBI taxonomy database was verified on the basis of an MD5 checksum. The FASTA search was performed with a cut-off/threshold value of E = 5000. In PCRv a maximum PCR product length of 5000 nucleotides (E= −5000) and the maximum number of permitted mismatches per primer/probe of 4 is set. A total of 49,094,040 sequences were searched in the NCBI nucleotide database, where 2,262,958 unique sequences were found. In these sequences, the primer and/or probe sequence were found at 4,435,964 different positions.

To check the analysis performed, FICS were inserted in all FASTA files on the basis of the PPRV PCR primers and probe being investigated. These FICS were inserted in 10-fold with an increasing number of mismatches to a maximum of 10. Those assays which achieve the highest mean FICS score for all 500 MB files were considered to offer the highest sensitivity.

The *in silico* sensitivity and specificity of the 6 assays designed in this paper, along with previously-published assays are shown in Table 1. All assays evaluated demonstrated an *in silico* specificity of 100% however, the 6 RT-qPCR assays we designed showed an *in silico* sensitivity ranging between 72.2%–99.1%. The M-gene assay showed the greatest *in silico* sensitivity however it yielded a low FICS score of 2.5. The F-gene assay demonstrated the greatest *in silico* performance (>98% sensitivity and greatest FICS performance) and was therefore chosen for further investigation. The F-gene assay showed a greater *in silico* sensitivity than the assays described by Polci et al., 2015 (90.6%), Batten et al., 2011 (90.5%) and Kwiatek et al., 2010 (58.6%). The RT-qPCR assay described by Batten et al., 2011 showed the greatest FICS score (5.7) which was followed by the F-gene RT-qPCR assay (4.6).

### 3.2 Diagnostic specificity and sensitivity

RNA dilution series of each of the 4 PPRV lineages were used to determine the diagnostic sensitivity of the F-gene RT-qPCR assay (Table 2). The RT-qPCR assay was found to detect PPRV over a 6 log_10_ dilution range (equating to between 10^0^ – 10^6^ TCID_50_  ml^−1^). The C_T_ values generated by both assays were significantly correlated (Pearsons correlation coefficient: r> 0.99, P < 0.001). Fifty eight samples submitted for PPRV diagnostic testing were included as the panel of samples to assess the F-gene RT-qPCR assay alongside the N-gene RT-qPCR assay. Out of 58 samples, 48 samples were positive using both RT-qPCR assays, an additional 2 samples were positive using the F-gene RT-qPCR assay and 8 samples were negative by both assays (Table 3). The two samples positive by the F-gene RT-qPCR assay yielded C_T_ values of 34.83 and 36.11. The mean C_T_ value difference between the N- and F-gene RT-qPCR assays was -0.15 which was not significant (t-test; P = 0.65). The F-gene RT-qPCR assay detected the PPRV vaccine strain (Nig 75/1) but did not yield C_T_ values when testing non-PPRV nucleic acid (supplementary data). In the samples obtained during an experimental infection study, both RT-qPCR assays showed full concordance (Table 4) and PPRV RNA was detected in EDTA blood samples at 4 dpi and then in all samples types by 6 dpi. At the first detection point, the mean C_T_ value difference between RT-qPCR assays was 0.07 in EDTA blood, 1.13 in ocular swabs and 0.85 in nasal swabs.

**Table 2:** C_T_ values for a**l**l PPRV lineages determined using the N-gene and F-gene RT-qPCR assays.

Dilution	Lineage I		Lineage II		Lineage III		Lineage IV	
	Ivory Coast/1997		Nigeria/1975		Dorcas U.A.E./1986		Georgia/Tbilisi/2016	
	N-gene	F-gene	N-gene	F-gene	N-gene	F-gene	N-gene	F-gene
	Mean C_T_ value (±SD)	Mean C_T_ value (±SD)	Mean C_T_ value (±SD)	Mean C_T_ value (±SD)	Mean C_T_ value (±SD)	Mean C_T_ value (±SD)	Mean C_T_ value (±SD)	Mean C_T_ value (±SD)
−1	19.58 (0.03)	19.88 (0.13)	19.46 (0.02)	20.37 (0.10)	16.85 (0.07)	18.48 (0.03)	18.15 (0.09)	19.38 (0.05)
−2	23.21 (0.13)	23.78 (0.20)	22.92 (0.04)	24.05 (0.16)	20.12 (0.10)	21.99 (0.06)	22.91 (0.04)	24.39 (0.07)
−3	26.48 (0.17)	27.23 (0.10)	26.76 (0.08)	27.93 (0.07)	23.77 (0.05)	25.79 (0.01)	24.98 (0.27)	26.95 (0.07)
−4	30.10 (0.16)	30.81 (0.07)	30.77 (0.08)	32.01 (0.15)	28.32 (0.14)	30.25 (0.35)	28.02 (0.27)	29.78 (0.09)
−5	34.22 (0.46)	35.17 (0.58)	33.98 (0.83)	35.04 (0.31)	32.19 (0.30)	34.18 (0.23)	30.30 (0.06)	31.98 (0.26)
−6	39.15 (3.70)	38.38 (1.73)	35.32 (n.d.)	38.16 (n.d.)	35.47 (1.15)	39.32 (0.48)	32.61 (0.58)	34.52 (0.34)

n.d. not determined due to insufficient number of replicates.

**Table 3:** Comparison of F-gene and N-gene RT-qPCR values.

Sample ID	Species	Matrix	N-gene	F-gene	Difference	Sample ID	Species	Matrix	N-gene	F-gene	Difference
			C_T_ value	C_T_ value	C_T_ value				C_T_ value	C_T_ value	C_T_ value
1-16-10	Caprine	Lung	24.70	25.61	−0.91	1-16-304	n.s.	Blood	Undet.	Undet.	n.a.
1-16-11	Caprine	Spleen	26.77	25.52	1.25	1-16-313	n.s.	Blood	32.47	31.87	0.60
1-16-12	Caprine	Intestine	31.16	29.91	1.25	1-16-315	n.s.	Blood	33.05	32.61	0.44
1-16-13	Caprine	Lymph	20.42	20.96	−0.54	1-16-317	n.s.	Blood	33.29	31.54	1.75
1-16-14	Caprine	Blood	30.75	31.54	−0.79	1-16-318	n.s.	Blood	Undet.	Undet.	n.a.
1-16-22	Caprine	Blood	29.86	30.95	−1.09	5-16-01	Ovine	Spleen	27.08	27.38	−0.3
1-16-23	Caprine	Blood	28.90	29.69	−0.79	5-16-02	Ovine	Lung	21.99	22.08	−0.09
1-16-26	Caprine	Blood	30.41	31.21	−0.80	5-16-03	Ovine	Spleen	28.83	29.48	−0.65
1-16-29	Caprine	Oral swab	24.72	25.38	−0.66	5-16-04	Ovine	Lung	30.61	31.19	−0.58
1-16-30	Caprine	Oral swab	30.63	30.56	0.07	5-16-05	Ovine	Blood	Undet.	36.11	n.a.
1-16-32	Caprine	Faecal swab	20.54	22.13	−1.59	5-16-06	Ovine	Spleen	27.59	28.59	−1
1-16-33	Caprine	Faecal swab	31.15	30.93	0.22	5-16-07	Ovine	Lung	28.67	29.26	−0.59
1-16-34	Caprine	Faecal swab	27.39	26.46	0.93	5-16-08	Ovine	Blood	33.07	33.05	0.02
1-16-35	Caprine	Oral swab	22.84	23.33	−0.49	5-16-09	Ovine	Spleen	28.88	29.51	−0.63
1-16-38	Caprine	Oral swab	25.05	25.66	−0.61	5-16-10	Ovine	Lung	Undet.	34.83	n.a.
1-16-50	Caprine	Blood	33.39	33.80	−0.41	1-19-01	Caprine	Swab	27.63	26.35	1.28
1-16-53	Caprine	Blood	30.09	30.60	−0.51	1-19-02	Caprine	Swab	Undet.	Undet	n.a.
1-16-54	Caprine	Blood	27.60	28.37	−0.77	1-19-03	Caprine	Swab	19.91	19.73	0.18
1-16-55	Ovine	Blood	32.96	32.43	0.53	1-19-04	Ovine	Swab	26.13	26.56	−0.43
1-16-56	Caprine	Blood	33.61	34.09	−0.48	1-19-05	Ovine	Swab	27.63	26.35	1.28
1-16-57	Caprine	Blood	35.50	36.64	−1.14	1-19-06	Caprine	Liver	25.87	21.73	4.14
1-16-59	Ovine	Blood	32.42	33.94	−1.52	1-19-07	Caprine	Ganglia	Undet.	Undet	n.a.
1-16-69	Caprine	Blood	Undet.	Undet.	n.a.	1-19-08	Caprine	Lung	Undet.	Undet	n.a.
1-16-82	Caprine	Blood	37.05	36.06	0.99	2-19-01	Ovine	Lung	23.24	22.53	0.71
1-16-84	Caprine	Blood	35.20	39.40	−4.20	2-19-02	Ovine	Lung	Undet.	Undet	n.a.
1-16-217	Caprine	Blood	26.37	27.27	−0.90	2-19-03	Ovine	Lung	Undet.	Undet	n.a.
1-16-218	Caprine	Blood	26.22	26.52	−0.30	2-19-04	Ovine	Lung	26.52	26.03	0.50
1-16-220	Caprine	Blood	32.54	31.41	1.13	2-19-05	Ovine	Lung	19.58	17.65	1.93
1-16-221	Caprine	Blood	27.64	28.16	−0.52	2-19-06	Ovine	Tongue	26.21	25.99	0.22

Undet. Undetected using RT-qPCR, n.a. not applicable, n.s. not specified.

**Table 4:** C_T_ values in samples obtained from experimentally-infected animals.

	dpi	EDTA blood		Ocular Swab		Nasal swab	
		N-gene C_T_ value	F-gene C_T_ value	N-gene C_T_ value	F-gene C_T_ value	N-gene C_T_ value	F-gene C_T_ value
Goat 1	0	Undet.	Undet.	Undet.	Undet.	Undet.	Undet.
	2	Undet.	Undet.	Undet.	Undet.	Undet.	Undet.
	4	29.91	28.03	39.60	37.05	Undet.	Undet.
	6	25.02	22.81	28.34	26.50	24.89	23.35
	8	27.66	25.10	24.90	23.13	18.98	18.62
Goat 2	0	Undet.	Undet.	Undet.	Undet.	Undet.	Undet.
	2	Undet.	Undet.	Undet.	Undet.	Undet.	Undet.
	4	27.57	28.12	21.97	22.68	26.60	26.68
	6	22.72	23.86	20.54	21.60	23.58	23.75
	8	23.04	25.56	19.60	19.78	17.68	18.91
Goat 3	0	Undet.	Undet.	Undet.	Undet.	Undet.	Undet.
	2	Undet.	Undet.	Undet.	Undet.	Undet.	Undet.
	4	27.57	27.81	31.83	29.92	36.20	35.48
	6	22.22	21.63	26.98	27.56	28.93	27.82
	8	23.01	23.64	22.35	22.02	23.33	22.62
Goat 4	0	Undet.	Undet.	Undet.	Undet.	Undet.	Undet.
	2	Undet.	Undet.	Undet.	Undet.	Undet.	Undet.
	4	28.86	29.68	35.12	34.36	26.52	25.31
	6	22.44	22.96	28.34	27.34	24.36	23.93
	8	24.55	24.96	23.18	22.28	20.47	20.80

Undet.: Undetected by RT-qPCR, N-gene: Batten et al., 2011, dpi: days post infection.

### 3.3 Analytical sensitivity, LOD and efficiency of the RT-qPCR assay

**Fig. 1:**
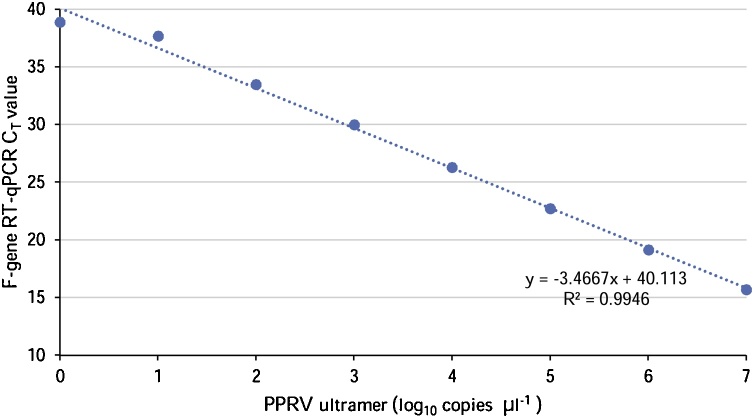
Standard curve for the F-gene RT-qPCR assay. Shown are the mean C_T_ values for Log dilutions of PPRV ultramer from 10^7^ copies μl^−1^ to 10^7^ copies μl^−1^. The limit of detection was 10 copies μl^−1^.

The relationship between C_T_ values and PPRV ultramer concentration was linear (Fig. 1) within the range of 10^7^ to 10^0^ copies μl^−1^ (R^2^≥ 0.99). The PCR efficiency estimated through the linear regression of the dilution curve (Ramakers et al., 2003) was calculated to be 94.3%, which is within the recommended range 90%–110%. The LOD was determined experimentally as 10 copies μl^−1^, which corresponds to the lowest concentration of the PPRV ultramer (10^1^ copies μl^−1^) for which C_T_ values were determined for all ten replicates.

## 4 Discussion

PPR is a significant disease of small ruminants that causes major economic losses in affected countries. Accurate and rapid diagnostics are critical for the control of PPR. The widespread use of RT-qPCR has the capacity to improve the diagnosis of PPR going forward and will contribute to the global eradication campaign. However, it is vital that the assays used are specific and sensitive. We considered that the most-widely used assays (some of which are specified in the OIE manual), were developed using the limited sequence data that was available at that time. The availability of high throughput sequencing platforms and the continued spread of PPR has resulted in additional sequences becoming available. This presented an opportunity to evaluate *in silico* sensitivity and specificity of existing assays and the necessity to develop new assays based on this additional sequence information. In this study we describe the design of an RT-qPCR assay for the detection of PPRV and evaluated its performance against i) clinical samples collected during an experimental infection study, ii) historical isolates of all 4 lineages and iii) samples from disease outbreaks.

Using the most up-to-date sequence information available, we performed an alignment to design a novel RT-qPCR assay. Although the PPRV N-gene is the most transcribed protein (Kumar et al., 2014), we decided to design a number of RT-qPCR assays targeting the 6 structural proteins to potentially identify an alternative conserved region, based on all full-genome PPRV sequences available in public domains. Crucial for the selection process, was a bioinformatics platform (PCRv) which integrated a number of freely-available software programs and allowed for an *in silico* assessment to be performed on each of the designed assays. This allowed for the elimination of potentially under-performing RT-qPCR assays which could not have been determined using the PCR assay design software in isolation. It should be noted that in silico analysis yields a preliminary estimation of assay sensitivity and specificity; therefore, a direct comparison of all published PPRV RT-qPCR assays using a well-characterised sample panel, will provide the only means of identifying the best-performing assay.

The incorporation of FICS into our *in silico* assessment allowed us to estimate the robustness of the designed RT-qPCR assays, rather than use the sequence data alone. The F-gene RT-qPCR assay showed the highest FICS score which indicates that the assay is the most robust to produce a specific amplicon even if the target sequence contains mismatches. As such, two of the assays designed (M-gene and P-gene) despite showing higher sensitivity and specificity than the F-gene RT-qPCR assay, were not considered for further evaluation. Notwithstanding, the selection criteria which we employed does not preclude the further use of the other RT-qPCR assays which we designed, either in a diagnostic role or to quantify specific PPRV genes.

Due to the widespread use of Sanger and next-generation sequencing technologies, a large number of PPRV partial and full-genome sequences are available in the public domain. For the F-gene RT-qPCR assay, this allowed for the inclusion of PPRV lineage III in the alignment, which was a limitation of the 4 most-widely used assays (Batten et al., 2011; Polci et al., 2015; Bao et al., 2008; Kwiatek et al., 2010). The PCRv software indicated a lower sensitivity for each of these assays in comparison with the F-gene RT-qPCR we developed. The assay described by Kwiatek et al., 2010 (demonstrating an *in silico* sensitivity of 58.6%) has been shown to offer poor sensitivity to PPRV lineage III strains in particular (Dr Arnaud Bataille and Dr Olivier Kwiatek, personal communication). The assay described by Adombi et al., 2011 showed the lowest in silico sensitivity (13.1%) of all PPRV RT-qPCR assays compared. This assay was originally used to detect PPRV in cell culture, however, no details concerning the sequences used to design the assay were indicated. The assay may have been designed specifically for the PPRV strain used in their study and thus may not have been intended for use in diagnostics. However, this assay has since been used in the development of an assay involving Loop-mediated isothermal amplification (LAMP) (Ashraf et al., 2017) where comparable performance was found between the LAMP assay and the RT-qPCR assay described by Adombi et al., 2011. The results from the *in silico* assessment performed using PCRv highlights the importance of RT-qPCR assay selection prior to undertaking work. Critical to the availability of up-to-date sequence data is the continued commitment of laboratories to deposit virus sequences in freely-accessible databases. This will allow for laboratories to perform an assessment of their in-use molecular assays using platforms such as PCRv in the confidence that they are utilising the most appropriate dataset for their evaluation. The continued use of poorly-performing RT-qPCR assays has the potential to overstate the performance of new/novel assays but more importantly, to yield false negative results for diagnostic samples. Clearly, it is important that the existing assays be evaluated based on the most up-to-date sequence information- the use of the PCRv platform described by van Weezep et al., 2019 provides a user-friendly means of doing so.

We performed the laboratory-based evaluation of the F-gene RT-qPCR assay alongside an assay targeting the PPRV N-gene (Batten et al., 2011) that is specified in the OIE terrestrial manual. The diagnostic sensitivity of the F-gene RT-qPCR assay was comparable to the N-gene RT-qPCR assay. This is interesting since an advantage of N-gene-based assays is that the N-gene is close to the promoter and hence is most abundant in infected cells (Kumar et al., 2014). The F-gene RT-qPCR assay was capable of detecting PPRV RNA from all PPRV lineages and in samples submitted to the OIE reference laboratory for PPR at The Pirbright Institute. The LOD of the RT-qPCR assay was 10 copies μl^−1^ which is in-line with detection limits specified for a number of other PPRV RT-qPCR assays; 10 copies μl^−1^ (Batten et al., 2011), 20 copies μl^−1^ (Polci et al., 2015) and 32 copies μl^−1^ (Kwiatek et al., 2010). The F-gene RT-qPCR assay demonstrated good efficiency (94.29%) since a PCR efficiency between 90% and 110% is generally considered acceptable (Broeders et al., 2014). In all, the F-gene RT-qPCR assay demonstrated excellent diagnostic and analytical sensitivity and provides an appropriate diagnostic assay for PPRV.

The F-gene RT-qPCR assay could be used as a valuable complementary tool to the existing RT-qPCR assays to rapidly confirm positive cases. In experimentally-infected goats, we found that the F-gene RT-qPCR could detect PPRV RNA at 4 dpi, when animals were still clinically unaffected. Thus, the F-gene RT-qPCR assay achieves sufficiently high sensitivity to detect PPRV in asymptomatic animals and is clearly suitable for surveillance purposes.

In conclusion, we have developed a novel, specific and sensitive RT-qPCR assay which can be used in isolation or in conjunction with OIE recommended assays for the detection of PPRV. This assay has been developed with consideration for newly-emerged PPRV strains and may provide an appropriate diagnostic assay for use during the global eradication campaign.

## Declaration of Competing Interest

The authors declare that they have no known competing financial interests or personal relationships that could have appeared to influence the work reported in this paper.
