# Supplementary material for: Improved PCR diagnostics using up-to-date in silico validation: An F-gene RT-qPCR assay for the detection of all four lineages of peste des petits ruminants virus
Source: J Virol Methods. 2019 Dec;274:113735. doi: 10.1016/j.jviromet.2019.113735 (PMC6853160; doi:10.1016/j.jviromet.2019.113735)
Supplement: Supplementary file 1 [file mmc1.docx]

**Supplementary Data**

C_T_ values for viruses tested using the F-gene RT-qPCR assay

| Virus (-and serotype) | F-gene RT-qPCR C_T_ value |
| --- | --- |
| BTV-1 | Undet. |
| BTV-2 | Undet. |
| BTV-3 | Undet. |
| BTV-4 | Undet. |
| BTV-5 | Undet. |
| BTV-6 | Undet. |
| BTV-7 | Undet. |
| BTV-8 | Undet. |
| BTV-9 | Undet. |
| BTV-10 | Undet. |
| BTV-11 | Undet. |
| BTV-12 | Undet. |
| BTV-13 | Undet. |
| BTV-14 | Undet. |
| BTV-15 | Undet. |
| BTV-16 | Undet. |
| BTV-17 | Undet. |
| BTV-18 | Undet. |
| BTV-19 | Undet. |
| BTV-20 | Undet. |
| BTV-21 | Undet. |
| BTV-22 | Undet. |
| BTV-23 | Undet. |
| BTV-24 | Undet. |
| BTV-26 | Undet. |
| BTV-27 | Undet. |
| BTV-29 | Undet. |
| Novel BTV serotype | Undet. |
| Morbillivirus PDV | Undet. |
| Morbillivirus MeV | Undet. |
| MAY 1/2017 FMDV Sample 1 | Undet. |
| MAY 1/2017 FMDV Sample 2 | Undet. |
| MAY 1/2017 FMDV Sample 3 | Undet. |
| MAY 1/2017 FMDV Sample 4 | Undet. |
| MAY 1/2017 FMDV Sample 5 | Undet. |
| Sheep Pox | Undet. |
| Goat Pox | Undet. |
| PPRV Nig75/1 vaccine strain | 25.91 |

*Undet. Undetected by RT-qPCR
